# Huxley’s Theory of Disease

**Published:** 1881-12

**Authors:** 


### Huxley's Theory of Disease.

The body is a machine of the nature of an army, not that of a watch or of a hydraulic apparatus. Of this army, each cell is a soldier, an organ a brigade, the central nervous system headquarters and field telegraph, the alimentary and circulatory system the commissariat. Losses are made good by recruits born in camp, and the life of the individual is a campaign, conducted successfully for a number of years, but with certain defeat in the long run. The efficacy of an army, at any given moment, depends on the health of the individual soldier, and on the perfection of the machinery by which he is led and brought into action at the proper time; and, therefore, if the analogy holds good, there can be only two kinds of diseases, the one dependent on abnormal states of the physiological units, the other on perturbation of their co-ordinating and alimentative machinery.—*Canadian Medical Record.*

